# A bile acid-related prognostic signature in hepatocellular carcinoma

**DOI:** 10.1038/s41598-022-26795-7

**Published:** 2022-12-26

**Authors:** Wang Zhang, Yue Zhang, Yipeng Wan, Qi Liu, Xuan Zhu

**Affiliations:** Department of Gastroenterology, Jiangxi Clinical Research Center for Gastroenterology, First Affiliated Hospital of Nanchang University, Nanchang, China

**Keywords:** Biomarkers, Gastroenterology, Oncology

## Abstract

Due to the high mortality of hepatocellular carcinoma (HCC), its prognostic models are urgently needed. Bile acid (BA) metabolic disturbance participates in hepatocarcinogenesis. We aim to develop a BA-related gene signature for HCC patients. Research data of HCC were obtained from The Cancer Genome Atlas (TCGA) and International Cancer Genome Consortium (ICGC) online databases. After least absolute shrinkage and selection operator (LASSO) regression analysis, we developed a BA-related prognostic signature in TCGA cohort based on differentially expressed prognostic BA-related genes. Then, the predictive performance of the signature was evaluated and verified in TCGA and ICGC cohort respectively. We obtained the risk score of each HCC patient according to the model. The differences of immune status and drug sensitivity were compared in patients that were stratified based on risk score. The protein and mRNA levels of the modeling genes were validated in the Human Protein Atlas database and our cell lines, respectively. In TCGA cohort, we selected 4 BA-related genes to construct the first BA-related prognostic signature. The risk signature exhibited good discrimination and predictive ability, which was verified in ICGC cohort. Patients were classified into high- and low-risk groups according to their median scores. The occurrence of death increased with increasing risk score. Low-risk patients owned favorable overall survival. High-risk patients possessed high immune checkpoint expression and low IC50 values for sorafenib, cisplatin and doxorubicin. Real-time quantitative PCR and immunohistochemical results validate expression of modeling genes in the signature. We constructed the first BA-related gene signature, which might help to identify HCC patients with poor prognosis and guide individualized treatment.

## Introduction

Worldwide, liver cancer ranks as the sixth most common cancer^1^. In Asia, the disease is the fifth most prevalent tumor^2^. More than 800,000 new cases of liver cancer are detected each year globally^3^, which reflects that liver cancer is a major global health problem. Cases of hepatocellular carcinoma (HCC) comprise approximately 90% of primary liver cancer cases^4^. The early diagnosis of HCC remains a challenge because of insidious and nonspecific symptoms at an early phase. Consequently, most patients are detected in advanced HCC with metastasis, which negates the opportunity for radical surgery and results in an unfavorable prognosis. Although tremendous advances have been achieved in the diagnosis and therapeutic strategy for HCC over the past decades, patients with HCC have an unsatisfactory prognosis. Therefore, developing and validating novel reliable prognostic models for HCC are of great importance for identifying patients with poor prognosis and making reasonable clinical decisions.

Bile acids (BAs) are the main constituents of bile. In the liver, cholesterol is converted into BAs through a series of chemical reactions. The human bile acid (BA) pool is composed of a large portion of primary BAs and a small portion of secondary BAs. Primary BAs are transformed into secondary BAs via deconjugation and 7α-dehydroxylation reactions with the help of gut microbiota^5^. Maintaining BA homeostasis is critical to normal liver function. In addition to their physiological role in intestinal lipid absorption, BAs function as endogenous signaling molecules that regulate multiple metabolic, inflammatory and immune processes^6–8^. Moreover, BAs are involved in the occurrence of multitudinous gastrointestinal carcinomas^9,10^. The biological processes of BA synthesis and metabolism are complicated and are regulated by various bile acid-related genes (BAGs). Consistent with BAs, BAGs are closely tied to the pathogenesis of HCC. SLC27A5, a fatty acid transporter-encoding gene, is essential for BA reconjugation^11^. SLC27A5 knockout facilitates lipid peroxidation and thereby contributes to NRF2/TXNRD1 pathway activation, which promotes HCC progression. This outcome indicates that SLC27A5 serves as a tumor suppressor^12^. FGF19 is a dietary-responsive endocrine hormone essential for enterohepatic circulation of BAs^13^. Abnormal expression of FGF19-FGFR4 has been shown to accelerate hepatocarcinogenesis and metastasis, while inhibition of the FGF19/FGFR pathway augments tumor-suppressive activity and improves the prognosis in patients with HCC^14,15^. In summary, the role of BAGs in hepatic carcinogenesis and prognosis is variable and worthy of further research.

Here, we developed the first BA-related prognostic signature based on these differentially expressed prognostic BAGs in HCC patients from The Cancer Genome Atlas (TCGA) cohort. In International Cancer Genome Consortium (ICGC) cohort, we verified the predictive performance of the signature. Additionally, we probed into the role of the signature in forecasting drug susceptibility.

## Materials and methods

### Data acquisition

Research data of HCC were obtained from TCGA (https://portal.gdc.cancer.gov) and ICGC (https://dcc.icgc.org/) online databases. In TCGA database, we obtained sequencing data of 374 HCC samples and 50 normal samples. After matching with clinical data, 370 HCC patients were included. In ICGC database, the transcriptome data and corresponding clinicopathological data of 232 HCC patients were obtained. BAGs were collected from the Gene Set Enrichment Analysis (GSEA) database (http://www.gsea-msigdb.org/gsea/index.jsp) using “bile acid” as the main search term, and 23 BA-related gene sets were found. These gene sets were comprised of genes that participate in BA biosynthesis, secretion, metabolism, and BA-related signaling pathways. After removing the overlapping genes, 199 BAGs were obtained. TCGA expression matrix did not contain the expression of MIR6886. Therefore, 198 BAGs were collected for further analysis and are displayed in Table S1. Figure S1 display the analysis flow of this study.

### Identification of differentially expressed prognostic BAGs

In TCGA cohort, we used p < 0.05, |log2 fold change (FC)|> 1 as standard to differentiate the differentially expressed BAGs between the normal group and the HCC group. The differentially expressed BAGs were displayed with a heatmap using “pheatmap” package in R4.1.2. Univariate Cox analysis was conducted to screen significantly prognostic BAGs after the expression data for the BAGs were combined with TCGA survival data. Subsequently, we intersected the differentially expressed BAGs with the prognostic BAGs to identify the differentially expressed prognostic BAGs for further analysis.

### Construction and validation of the gene signature

Based on the differentially expressed prognostic BAGs, we developed a risk model by using least absolute shrinkage and selection operator (LASSO) regression analysis. After LASSO regression analysis, 4 differentially expressed prognostic BAGs and its regression coefficients were obtained and were chosen for model building. Then, patient risk score was calculated with the following equation: Risk score = (Expression value of BAG1) * Coefficient 1 + (Expression value of BAG2) * Coefficient 2 + … + (Expression value of BAGn) * Coefficient n. According to the median risk score, we divided HCC cases into high- and low-risk groups. The discrimination ability of the signature was evaluated by principal component analysis (PCA). We utilized the area under the curve (AUC) to evaluate the prediction ability of gene signature.

### Functional enrichment analysis

GSEA, Gene Ontology (GO) and Kyoto Encyclopedia of Genes and Genomes (KEGG) analyses were performed with the “clusterProfiler” package in R4.1.2. To evaluate the infiltration of various immune cells and the activity levels of diverse immune-related pathways in each HCC sample, we conducted single-sample gene set enrichment analysis (ssGSEA).

### Tumor mutation burden and drug sensitivity analysis

Tumor mutation burden (TMB) scores were calculated and gene mutations were visualized via the “Maftools” package, which is easy to use and contains multiple statistical and computational approaches for cancer genome research^16^. According to patient gene expression data, the clinical chemotherapeutic response of patient can be accurately predicted by using the “pRRophetic” package^17^. The semi-inhibitory concentration (IC50) is a useful indicator of drug sensitivity and refers to the drug concentration required for 50% cell proliferation suppression in vitro. Therefore, a high IC50 indicates a low sensitivity of neoplastic cells to the drug. We predicted the drug sensitivities of HCC patients by using the pRRophetic package.

### Cell culture and real-time quantitative PCR analysis (RT–qPCR)

Human hepatocyte cell line LO2 and HCC cell line HepG2 were maintained in RPMI 1640 (Gibco, USA) and DMEM (DMEM, Gibco, USA), respectively. Complementary DNA was obtained by reverse transcription of total cellular RNA with a TIANScript RT kit (Tiangen, China). Quantitative PCR was carried out with 2× M5 HiPer UltraSYBR Mixture (Mei5bio, China). GAPDH was served as an internal control. PCR primers are displayed in Table S2.

### Statistical analysis

R4.1.2 software and SPSS 20.0 were applied to statistical analyses. P < 0.05 was considered statistically significant.

## Results

### Clinicopathological features of the included HCC patients

We initially analyzed the clinicopathological features of the HCC patients in this study. In TCGA cohort, 370 HCC patients with clinicopathological data were used for model building and survival analysis. 232 HCC patients derived from ICGC database were served as a validation cohort. The Clinicopathological features of HCC patients in this study are shown in Table 1. In TCGA and ICGC cohorts, most patients were male, with older than 60 years, no family history. In TCGA cohort, part of HCC patients had liver fibrosis or cirrhosis (137/370, 37.03%). The main HCC risk factor in medical history is alcohol intake.

**Table 1:** Clinicopathological features of HCC patients in this study.

Variables	TCGA cohort	ICGC cohort
**Survival status**		
Alive	244	189
Dead	126	43
**Age (year)**		
< 60	169	45
≥ 60	201	187
**Gender**		
Female	121	61
Male	249	171
**Stage**		
I	171	36
II	85	106
III	85	71
IV	5	19
Unknown	24	
**Family history**		
Positive	112	74
Negative	207	143
Unknown	51	15
**Grade**		
I	55	
II	177	
III	121	
IV	12	
Unknown	5	
**Fibrotic Ishak score**		
0	74	
1–4	59	
5–6	78	
Unknown	159	
**HCC risk factors in medical history**		
Alcohol	125	
Viral hepatitis	112	
Alcohol & viral hepatitis	42	
Other^a^	27	
Unknown	64	
**Prior malignancy**		
Positive		30
Negative		202

^a^Other risk factors include Gibert’s syndrome, hemochromatosis, medicine and so on.

### Identification and functional enrichment analysis of the differentially expressed BAGs

**Figure 1:**
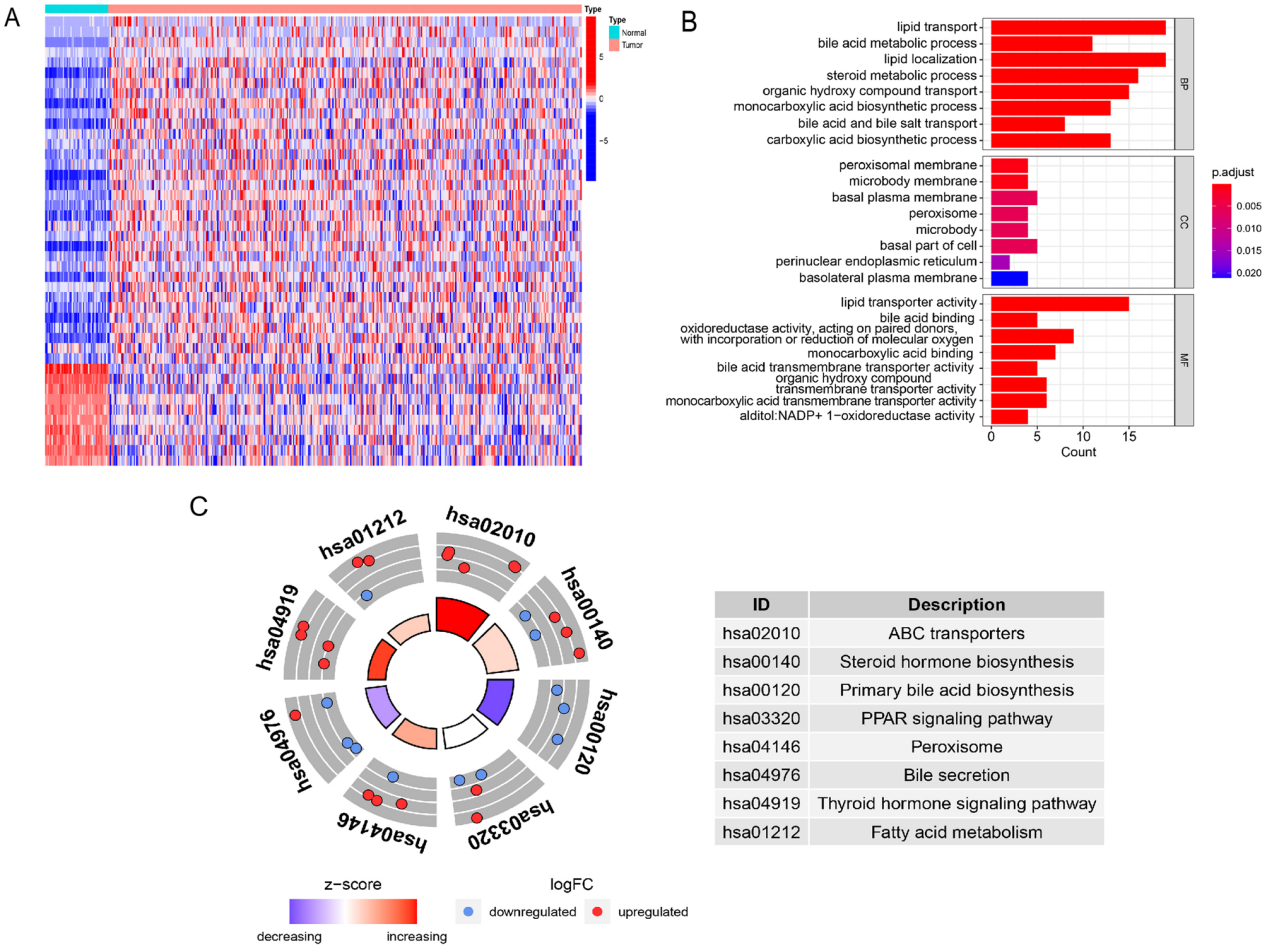
Differentially expressed BAGs and functional enrichment analyses based on TCGA cohort. (**A**) Heatmap of differential BAGs. The heatmap was created by using “pheatmap” package in R4.1.2 (https://www.r-project.org/). (**B**) GO analysis of differential BAGs. (**C**) KEGG analysis of differential BAGs.

Before constructing the prognostic signature, we screened the differentially expressed BAGs and analyzed the potential roles of these genes in hepatocarcinogenesis. 198 BAGs obtained from 23 BA-related gene sets were included in the differentially expressed analysis of TCGA cohort. We identified 44 genes as differentially expressed BAGs between the normal and tumor groups (Table S3). As shown in Fig. 1A, most of the differentially expressed BAGs were upregulated in the HCC samples. To explore the potential mechanism by which these genes contribute to HCC, GO and KEGG analyses were carried out. According to biological process (BP) analysis, Steroid metabolism, BA metabolism, BA and bile salt transport were significantly enriched. As indicated by cellular component (CC) analysis, these differentially expressed BAGs were significantly concentrated in peroxisomes and microbodies. Lipid, BA and monocarboxylic acid transmembrane transporter activity were significantly enriched categories according to GO molecular function (MF) analysis (Fig. 1B). KEGG (https://www.kegg.jp/), an integrated database, establishes the connection between genomic information and higher order functional information, which contributes to the functional annotation of genes and proteins^18,19^. KEGG analysis was carried out to explore the pathway enrichment of differentially expressed BAGs, which is useful for understanding the functions of the genes. As indicated by KEGG analysis, the pathways involved with primary BA biosynthesis and bile secretion were enriched (Fig. 1C).

### Construction of the BA-related risk signature in TCGA cohort

**Figure 2:**
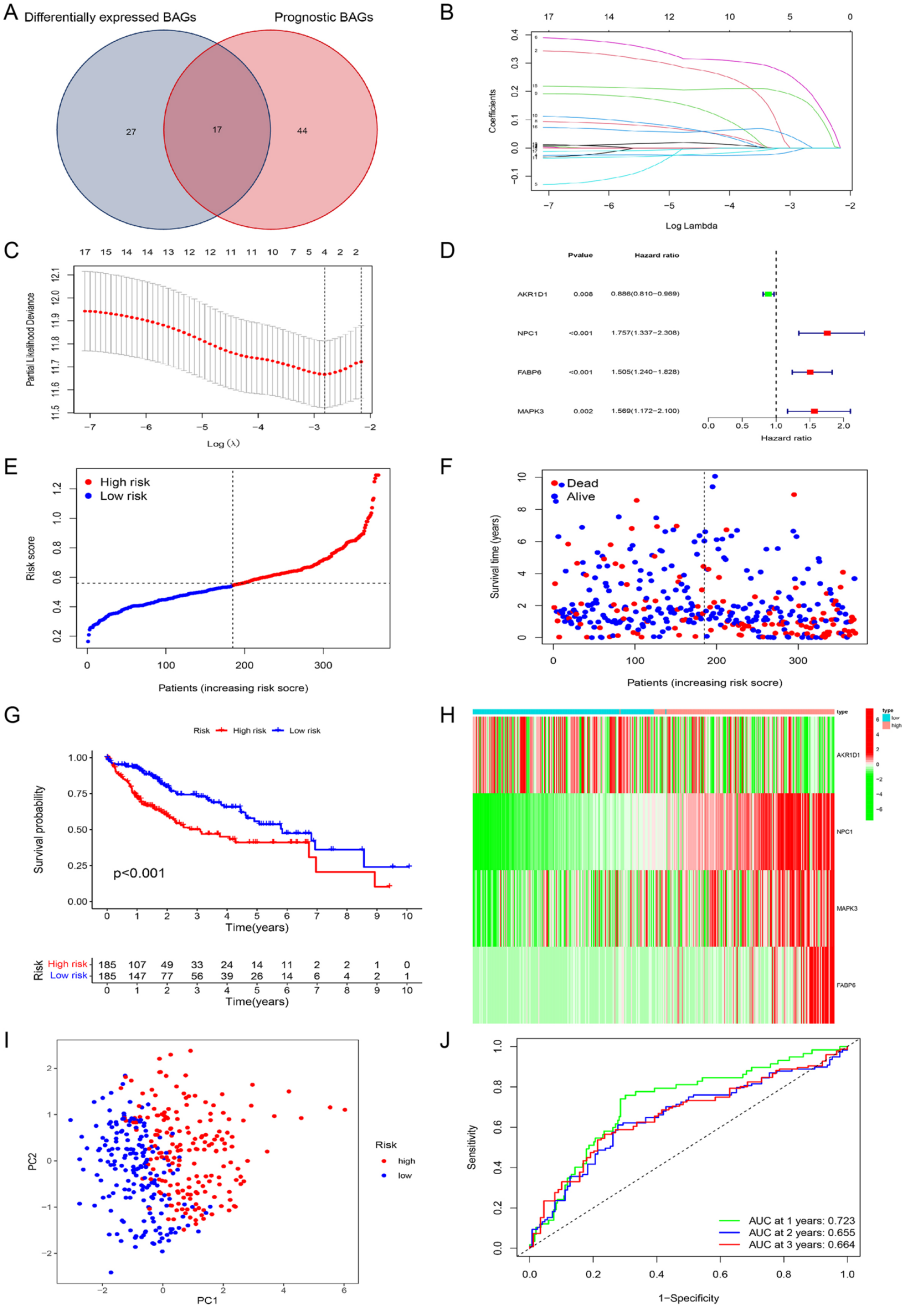
Construction of the bile acid-related risk signature in TCGA cohort. (**A**) Venn diagram displaying 17 intersecting genes between the 44 differentially expressed BAGs and 61 prognostic BAGs. (**B**) LASSO regression analysis of 17 differentially expressed prognostic BAGs. (**C**) Cross-validation of LASSO regression analysis. (**D**) Forest plot of modeling genes. (**E**) Risk grouping based on median risk value. (**F**) Relationship between risk value and survival status. (**G**–**I**) KM curve, expression of modeling genes and PCA based on risk grouping. (**J**) ROC curves in TCGA cohort.

In order to constructed a BA-related risk signature in TCGA cohort, the prognostic BAGs were first screened by univariate Cox analysis from 198 BAGs with the criteria of P < 0.05. We identified 61 genes as significantly prognostic BAGs, and these genes are displayed in Table S4. Subsequently, 17 differentially expressed prognostic BAGs were obtained by intersecting the 44 differentially expressed BAGs with the 61 prognostic BAGs (Fig. 2A). We subjected the common differentially expressed genes (DEGs) that we have obtained from intersection of differentially expressed BAGs with the prognostic BAGs to feature selection—LASSO regression method to select only those genes which are very relevant to formulate a bile acid-related prognostic signature (Fig. 2B and C). Finally, 4 BAGs were selected for model building. The 4 BAGs and its regression coefficients are displayed in Table S5. The correlation network indicated that AKR1D1 had a negative relationship with other modeling genes (Fig. S2). As shown in Figs. 2D and S3A–3D, AKR1D1 was protective factor for overall survival, while the others were risk factors based on the univariate Cox analysis. In addition, AKR1D1 was upregulated, while the others were downregulated in the normal samples (Fig. S4A–4D). Based on the 4 modeling genes and its regression coefficients, the equation for calculating the patient risk score was obtained and was as follows: Risk score = (Expression value of AKR1D1)*(− 0.0045) + (Expression value of NPC1) * 0.2253 + (Expression value of FABP6) * 0.1733 + (Expression value of MAPK3) * 0.0230. In TCGA cohort, patients were classified into high- and low-risk groups according to their median scores (Fig. 2E). The occurrence of death tended to increase with increasing risk score, which was further confirmed by survival analysis (Fig. 2F, G). The two groups were different in the expression of the modeling genes (Fig. 2H). The risk signature exhibited good discriminability and predictive ability (Fig. 2I and J).

### Validation of the BA-related risk signature in ICGC cohort

**Figure 3:**
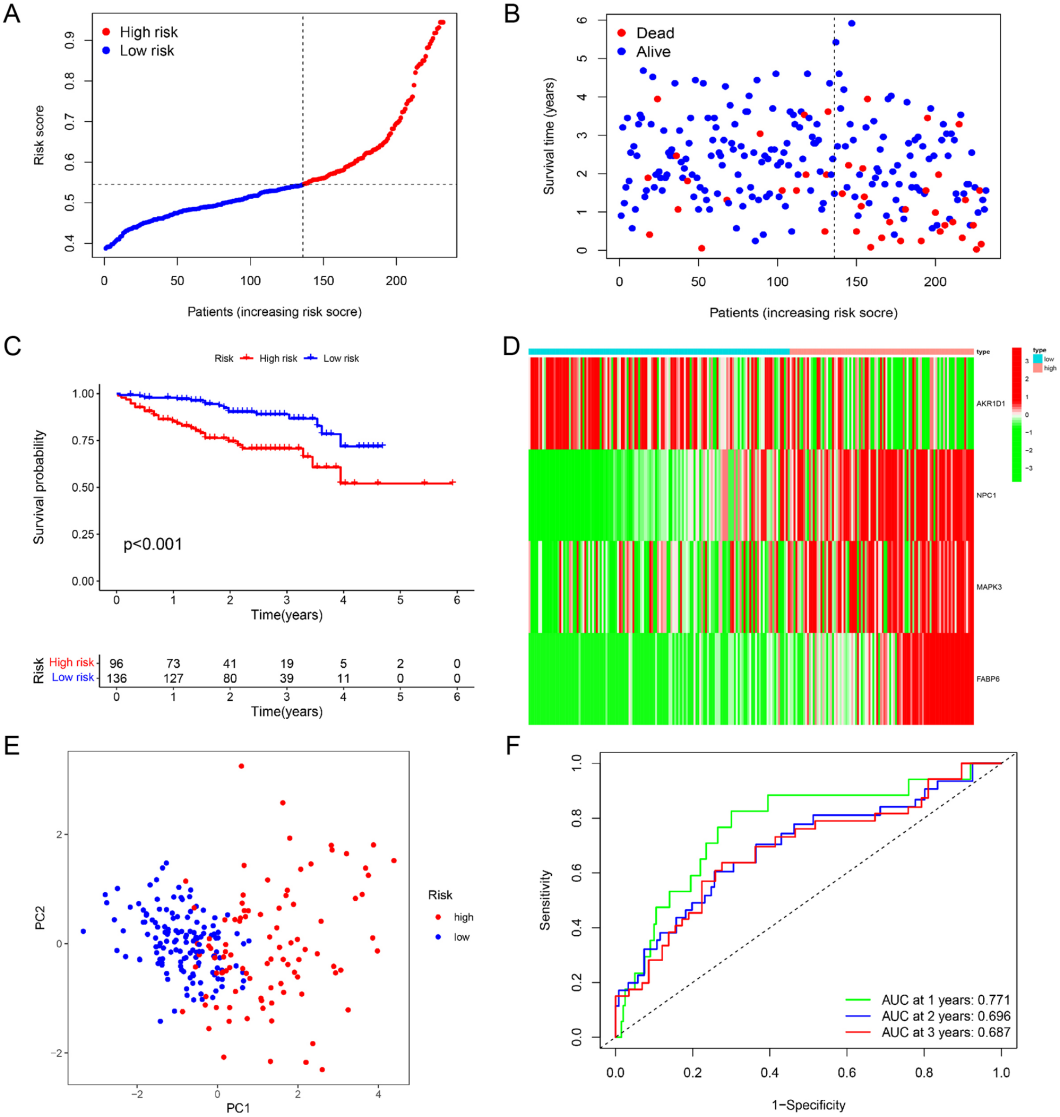
Verification of the bile acid-related risk signature in ICGC cohort. (**A**) Risk grouping based on TCGA median risk value. (**B**) Correlation between risk value and survival status. (**C**–**E**) KM curve, expression of modeling genes and PCA based on risk grouping. (**F**) ROC curves in ICGC cohort.

The model possessed good predictive performance in TCGA cohort. We then verified the predictive performance of the signature in external validation cohort. In ICGC cohort, we obtained patient risk scores with the above equation. Then, high- and low-risk patients were distinguished with the same median scores (Fig. 3A). Figure 3B shows an increase in mortality with increasing risk score. Kaplan–Meier (KM) plot further confirmed that high risk value hinted unfavorable overall survival (Fig. 3C). In high-risk patients, increased levels of the modeling genes were observed except AKR1D1 (Fig. 3D). The signature also displayed good differentiating capacity and predictive power in the external validation cohort (Fig. 3E and F).

### Independent predicting power of BA-related model

**Figure 4:**
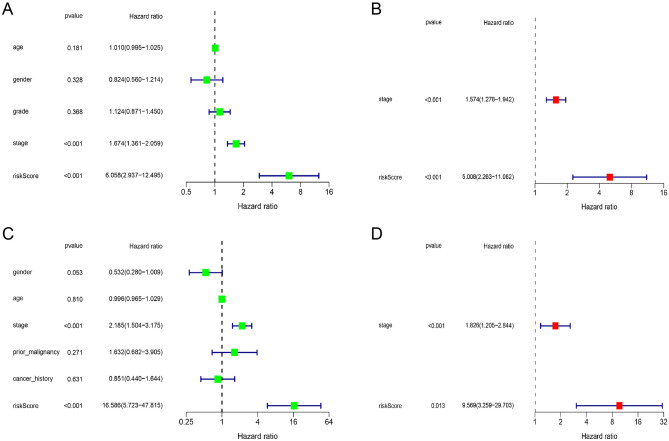
Independent predicting power of BA-related model. (**A**, **B**) Forest plots of Cox regression analysis results in TCGA cohort. (**C**, **D**) Forest plots of Cox regression analysis results in ICGC cohort.

We subsequently explored whether the risk score was independent prognostic factor for HCC patients. After merging risk score with clinicopathological characteristics, independent prognostic factors were identified with Cox regression analysis. The prognosis of TCGA and ICGC patients was impacted by risk score and tumor stage, which revealed by univariate Cox regression analysis in Fig. 4A and C. Risk score manifested independent predicting power for unfavorable survival, which corroborated by multivariate Cox regression analysis in Fig. 4B and D.

### Risk score-related clinicopathological features

**Figure 5:**
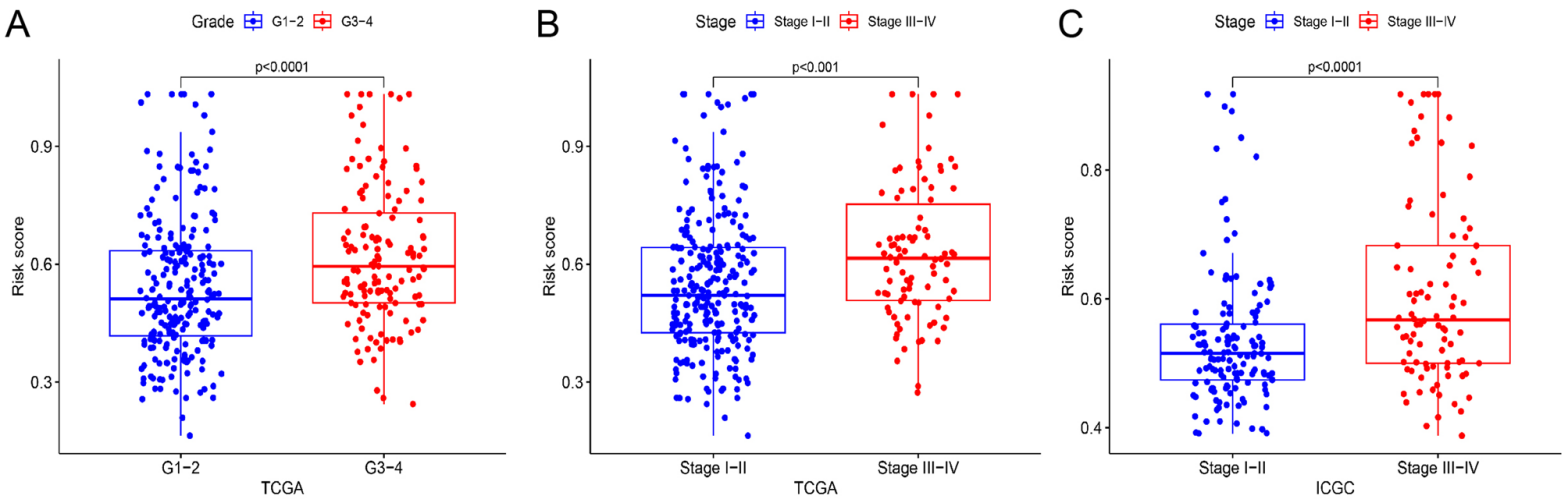
Risk score-related clinicopathological features. (**A**, **B**) Grade and stage in TCGA cohort. (**C**) Stage in ICGC cohort.

In order to understand the correlation between the model and clinicopathological features, we explored the discrepancy in risk score among patients with different clinicopathological features in TCGA and ICGC cohorts. We discovered risk score increased with increasing HCC severity. In TCGA cohorts, patients grading severity aligned with risk score (Fig. 5A). High risk scores were observed in patients with stage III–IV (Fig. 5B and C). Other clinicopathological parameters, including age, gender, prior malignancy and cancer history, did not correlate significantly with the risk score (Fig. S5A–5F).

### Functional enrichment analyses based on risk score

**Figure 6:**
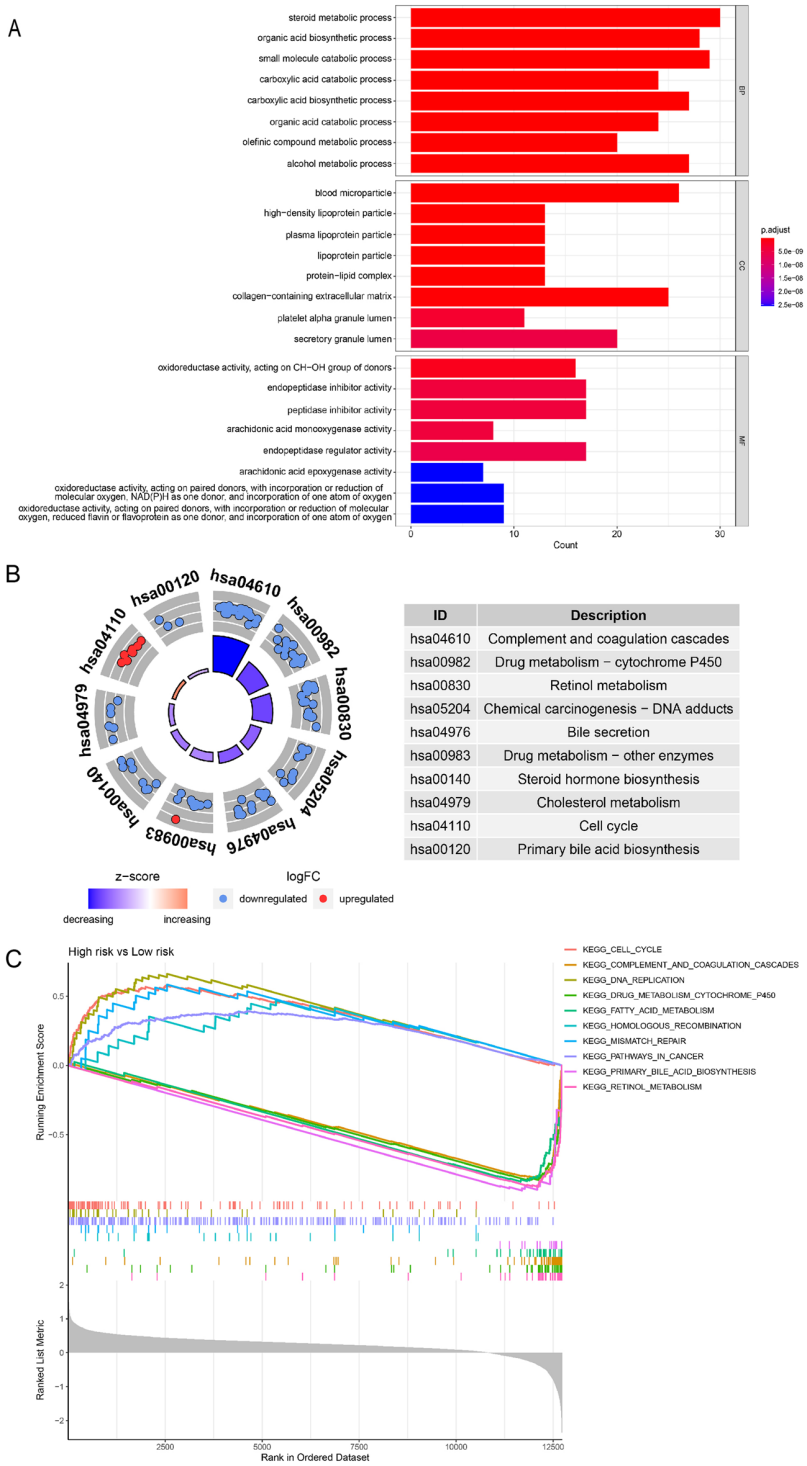
GO and KEGG analyses and GSEA based on risk grouping in TCGA cohort. (**A**–**C**) Results of GO analysis, KEGG analysis and GSEA.

According to the previous results, patient outcome varies with risk score. Whereafter, we conducted functional enrichment analyses to explore the possible mechanisms that contributed to this phenomenon. Based on risk grouping, we screened 219 genes as differential genes with the criteria of P < 0.05 and |log2FC|> 1 (Table S6). Subsequently, we conducted GO and KEGG enrichment analyses with clusterProfiler package. According to BP analysis, organic acid and steroid metabolism was significantly enriched. As indicated by CC analysis, these genes were significantly centralised in lipoprotein particles and protein-lipid complexes. In MF analysis, oxidoreductase activity and arachidonic acid monooxygenase activity were enriched (Fig. 6A). According to KEGG analysis, the pathways linked with complement and coagulation cascade, chemical carcinogenesis and bile secretion were concentrated (Fig. 6B). The pathways correlated with cell cycle and tumorigenesis were upregulated while the pathways correlated with drug metabolism and primary BA biosynthesis were downregulated in high-risk patients, as revealed by the GSEA (Fig. 6C).

### Risk score-related immune status

**Figure 7:**
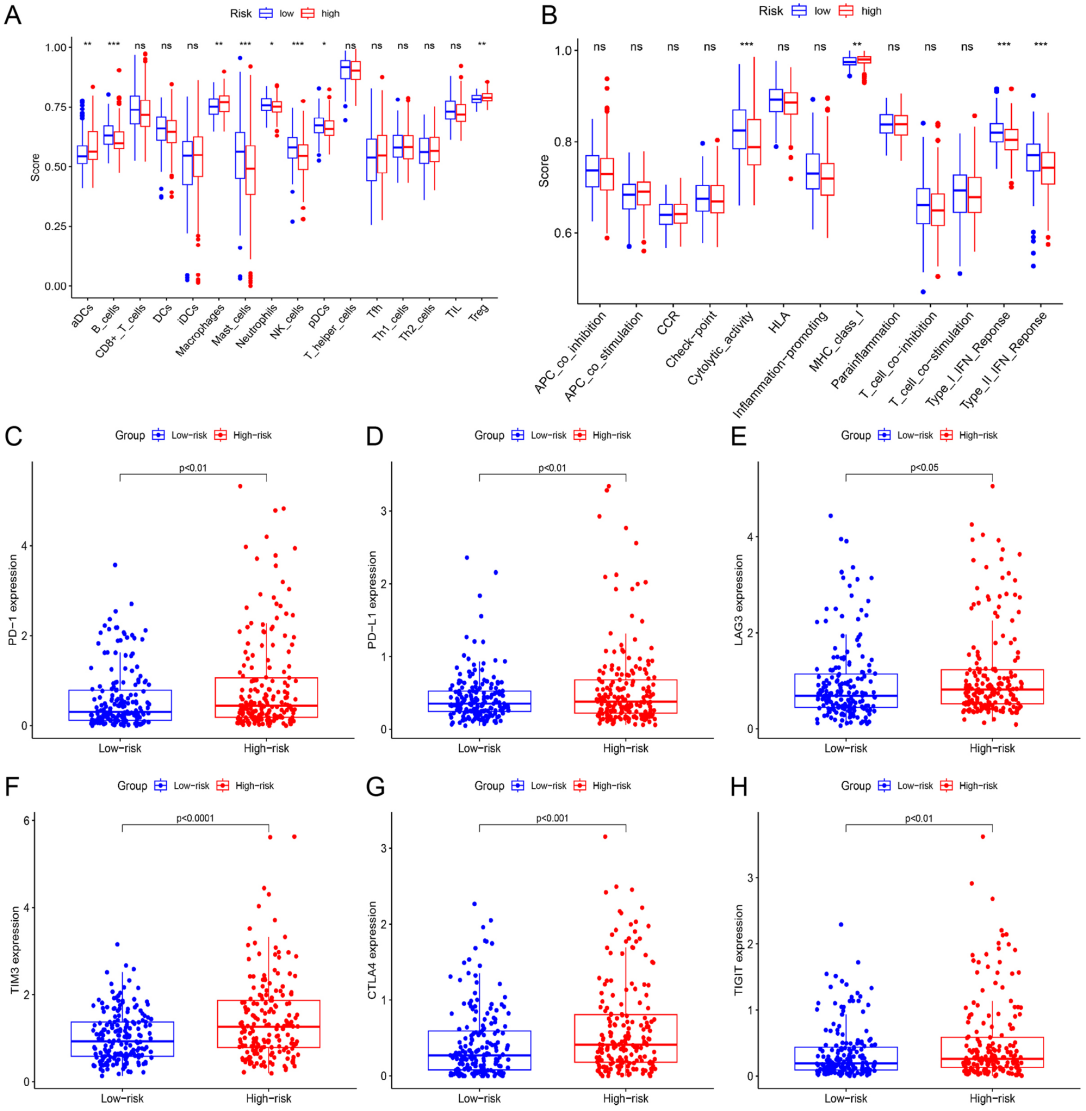
Risk score-related immune status. (**A**, **B**) Results of ssGSEA. (**C**–**H**) immune checkpoint expression based on risk grouping. *P < 0.05, ** P < 0.01, *** P < 0.001, ns, not significant.

The immune microenvironment plays an essential role in hepatocarcinogenesis, therefore, we conducted ssGSEA to evaluate the immune status. In high-risk patients, the proportions of activated dendritic cells (aDCs), macrophages and Tregs were increased, while the infiltration of natural killer (NK) cells exhibited the opposite trend (Fig. 7A). In low-risk patients, high scores of cytolytic activity and IFN response were observed (Fig. 7B). Patients with high immune checkpoint expression benefit more from immune checkpoint inhibitor therapy. Therefore, we evaluated the immune checkpoint expression in HCC patients. In our study, High-risk patients owned high immune checkpoint expression (Fig. 7C–H).

### Differences in TMB and drug sensitivity based on risk grouping

**Figure 8:**
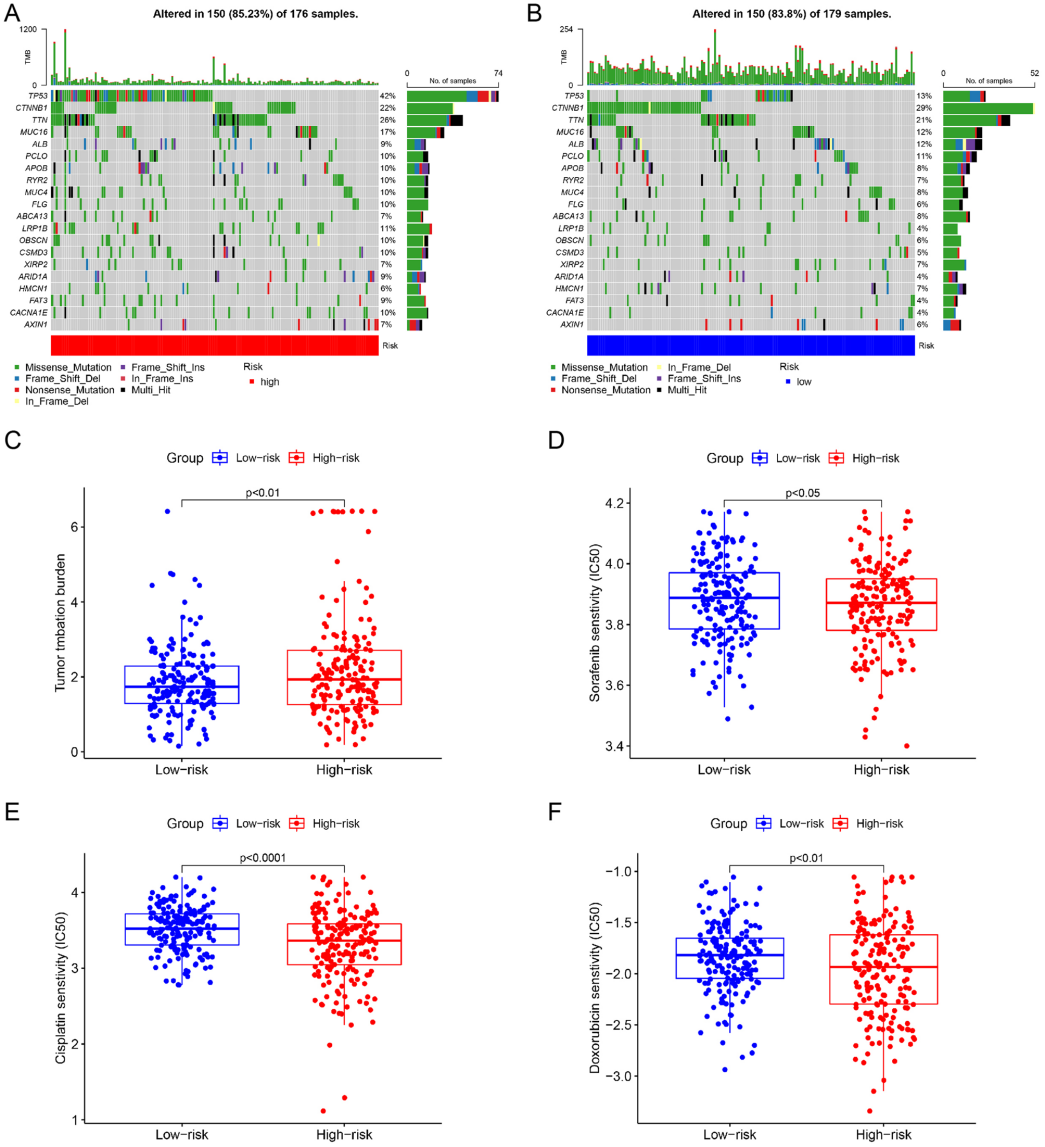
Differences in TMB and drug sensitivity based on risk grouping. (**A**, **B**) Waterfall plots of mutant genes. (**C**) TMB based on risk grouping. (**D**–**F**) The sensitivity comparisons of sorafenib, cisplatin and doxorubicin based on risk grouping.

TMB reflects the quantity of the somatic mutation in tumor tissues, which has a close relationship with response to immunotherapy and is considered as a promising immune-response biomarker^20,21^. Therefore, we assessed the TMB in HCC patients with different risk scores. Top 20 mutated genes were visualized by waterfall plots (Fig. 8A and B). TP53 is the most mutated gene in high-risk group. The mutation frequency of TP53 in high-risk group is 42% and the main mutation type is missense mutation. In low-risk group, CTNNB1 is the most frequently mutated gene and its mutation type is predominantly missense mutation. High-risk patients tend to have higher TMB (Fig. 8C). Drug resistance not only influences the therapeutic effect but also shortens patient survival. The prediction of drug sensitivity is a vital component in individual treatment. Thus, we evaluated the difference in drug sensitivity based on risk grouping. In high-risk patients, we found significantly low IC50 values for sorafenib, cisplatin and doxorubicin (Fig. 8D–F), which meant these drugs were more effective for these patients. The high- and low-risk patients had same sensitivity to mitomycin (Fig. S6).

### Validation of the modeling genes

**Figure 9:**
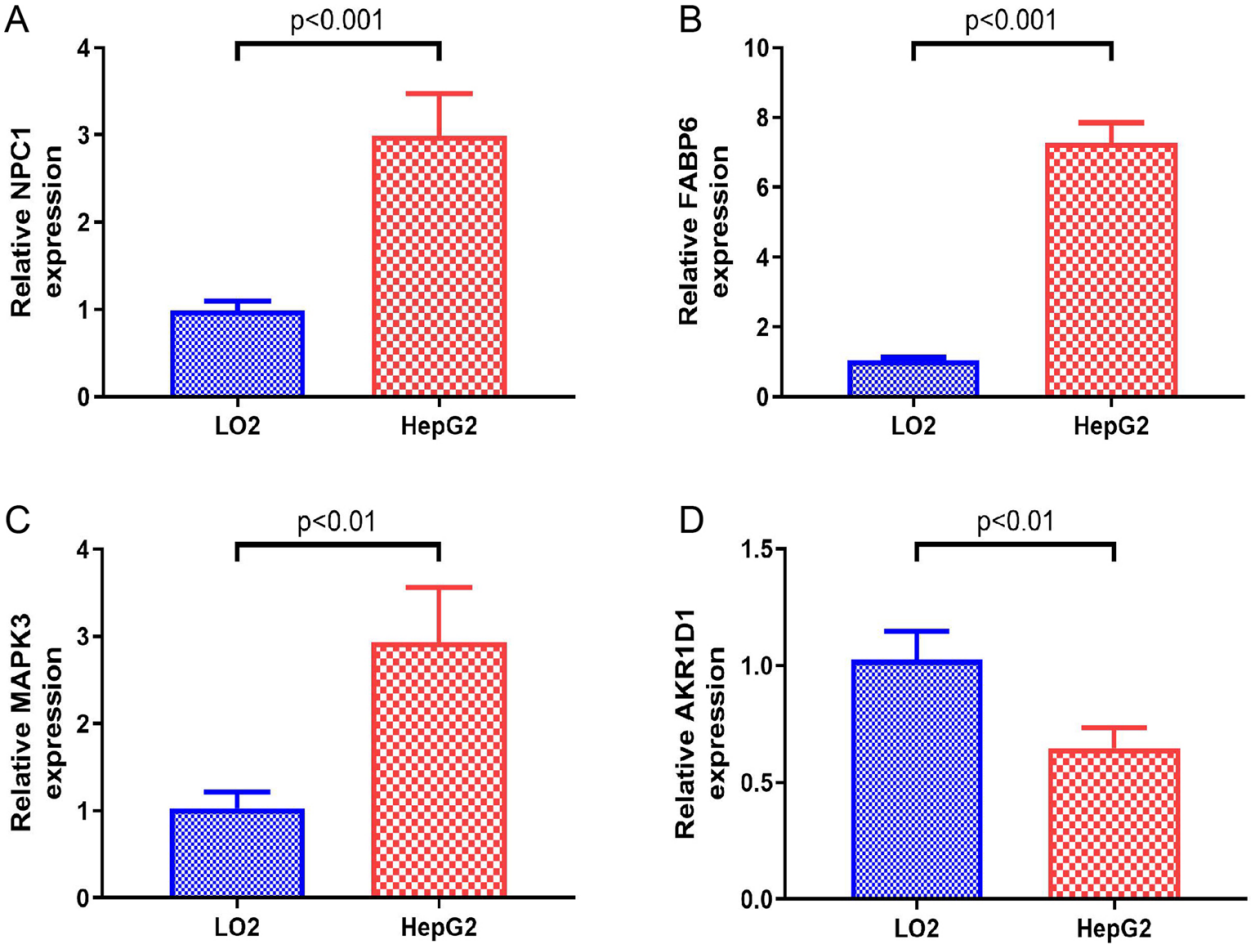
Verification of modeling genes in our cell lines. (**A**) NPC1, (**B**) FABP6, (**C**) MAPK3, (**D**) AKR1D1.

According to the previous analyses, modeling genes are expressed differently in normal and tumor samples. We subsequently validated the protein and mRNA levels of the modeling genes in the Human Protein Atlas database and our cell lines, respectively. Compared with hepatocyte cell line LO2, HCC cell line HepG2 had higher mRNA expression levels of NPC1, FABP6 and MAPK3, which was consistent with the bioinformatic analysis results (Fig. 9A–C). Elevated mRNA levels of AKR1D1 were verified in LO2 cells (Fig. 9D). In the Human Protein Atlas database, we verified the protein expression of 4 modeling genes. High levels of MAPK3 protein expression were observed in HCC samples, while AKR1D1 protein expressions were upregulated in normal tissues. FABP6 was not detected in normal or HCC samples (Fig. S7A–D).

## Discussion

The pathogenesis of HCC is extremely complicated and not fully understood. BAGs not only regulate the synthesis and metabolism of BAs but also play important roles in hepatocarcinogenesis. At present, most research focuses on the effect of a single BA gene on the biological behavior and prognosis of HCC. Considering the multiple impacts of BAGs on hepatocarcinogenesis, we constructed the first prognostic signature of BAGs to distinguish high-risk patients and guide personalized treatment.

44 differentially expressed BAGs were identified between normal and HCC samples based on 198 BAGs collected from 23 BA-related gene sets. Unsurprisingly, these genes were significantly concentrated in BA synthesis metabolism, which confirmed by functional enrichment analyses. Then the first BA-related prognostic signature was constructed based on 4 differentially expressed prognostic BAGs after a systematic analysis. Subsequently, we calculated patient risk scores with this signature. The risk signature exhibited good discriminability and predictive ability according to PCA, receiver operating characteristic (ROC) and KM curve analysis. Risk score manifested independent predicting power for unfavorable survival. In addition, we validated not only the predictive value and stability of genetic model but also the expression levels of modeling genes.

The model we constructed contained 4 differentially expressed prognostic BAGs, namely, NPC1, FABP6, MAPK3 and AKR1D1. NPC1 encodes the membrane protein Nieman-Pick C1 (NPC1), which is critical for cholesterol export from lysosomes/late endosomes^22^. NPC1 mutation results in a life-limiting lysosomal storage disease, Niemann-Pick disease type C, and increases the risk of hepatocarcinogenesis^23,24^. FABP6 is a BA transporter in ileal epithelial cells and is critical for the enterohepatic circulation of BAs^25^. FABP6 also participates in the progression of numerous types of cancer^26–28^. Compared with adjacent normal liver tissues, overexpressed FABP6 was observed in HCC tissues^29^. Consistent with this, we obtained similar results in our study. However, the specific mechanisms of FABP6 in HCC need to be further studied. MAPK3, also known as extracellular signal-regulated kinase 1 (ERK1), is a member of the MAPK signaling pathway, which participates in tumorigenesis and metastasis in multiple tumor types^30^. ERK1, together with ERK2, plays an important role in BA metabolism. BAG expression profile was altered in ERK1/2 knockout mice. ERK1/2 knockout decreased the expression of BA uptake genes and increased the expression of BA export gene^31^. CYP7A1 serves as a rate-limiting enzyme to participate in BA biosynthesis^32^. In human hepatocytes, ERK1/2 negatively regulated the expression of CYP7A1 and fibroblast growth factor 19 inhibited CYP7A1 expression partially through activation of ERK1/2^33^. In the pathogenesis of HCC, ERK1, but not ERK2, phosphorylated intestine-specific homeobox, resulting to its nuclear translocation and the expression of downstream genes related to cell proliferation, malignant transformation and the resistance to sorafenib^34^. The expression and activation of MAPK3 were upregulated in HCC tissues and cells^35,36^. In line with this, our results indicated that MAPK3 were highly expressed in HCC. In addition, MAPK3 was risk factors for overall survival. Delta(4)-3-Ketosteroid 5-Beta-Reductase encoded by AKR1D1 acts as one of the key reductases related to BA biosynthesis^37^. Aberrant expression of AKR1D1 contributes to BA synthesis defect, metabolic disorders and liver failure^38–40^. In human hepatoma cells, AKR1D1 regulated glucocorticoid clearance and the activation of the glucocorticoid receptor^41^. Overexpression of AKR1D1 significantly inhibited the cell viability and the activation of androgen receptor signaling pathway in HCC cell^42^. Low expression of AKR1D1 was observed in HCC tissue^43^. Here, HCC patients with elevated AKR1D1 had favorable prognosis, indicating that AKR1D1 might have an antitumor effect in HCC. AKR1D1 also had a good diagnostic ability for HCC^42^. Our model might be useful to guide the diagnosis of HCC and the genes included in our model might serve as promising diagnostic biomarkers for HCC. Further studies are warranted.

Tumor immune microenvironment influences hepatocarcinogenesis and treatment strategies for patients^44^. BAs not only play an essential role in intestinal lipid absorption but also modulate immunity^6,45^. Therefore, we conducted ssGSEA to evaluate the immune status. In high-risk patients, the proportions of aDCs, macrophages and Tregs were increased, while the infiltration of NK cells exhibited the opposite trend. In low-risk patients, high score of IFN response was observed. In HCC patients, Treg intratumoral accumulation triggered by intratumoral macrophages suppresses tissue-derived CD4+ CD25− T cells activation, which contributes to HCC progression and unfavorable prognosis^46^. In addition, Tregs inhibit CD8+ T cells proliferation and activation in HCC patients^47^. NK cells form nearly half of the liver's lymphocytes. In HCC microenvironment, a high abundance of NK cells in HCC tissue is a favorable factor for survival^48^. Via a STAT3-dependent pathway, IFNs induce HCC cell apoptosis through blocking β-catenin signaling pathway^49^. Impaired antitumor immunity may contribute to adverse prognosis in high-risk patients.

Immune checkpoint inhibitors (ICIs) are new and effective treatment choices for HCC patients. However, as with other drugs, not all patients are similarly sensitive to ICIs. Distinguishing the ICI-sensitive population is important to individualized therapy. The interaction of immune checkpoint proteins with their ligands leads to T cells inactivation and immunosuppression. As monoclonal antibodies, ICIs can block the process by binding with immune checkpoint proteins, thus restoring T cell activation and immune attack^50^. ICIs might have better effects on high-risk patients who have upregulated expression levels of common immune checkpoint. TMB is a significant indicator of genomic stability^51^. Tumor with high TMB can generate more neoantigens, some of which can serve as signals to induce immune system activation and T-cell reactivity, thus increasing sensitivity of tumor cells to immunotherapy^52,53^. Therefore, TMB is an emerging biomarker for predicting ICIs response, and ICIs possess better effects in patients with a high TMB^54^. In our study, high TMB was discovered in high-risk patients. Chemotherapy is an important component in comprehensive treatment of advanced HCC. Predicting chemotherapeutic drug sensitivity is of great significance to individualized therapy. The gene expression may act as a surrogate for unmeasured phenotypes that are directly relevant to chemotherapeutic sensitivity, which provides the possibility to predict chemotherapeutic sensitivity based on gene expression^55^. Many researchers use gene expression data to predict drug sensitivity, including chemotherapeutic agents^56,57^. In our study, we evaluated the sensitivity to some chemotherapeutic and targeted drugs in HCC patients. Our results revealed that low IC50 values for sorafenib, cisplatin and doxorubicin were observed in high-risk patients, which indicated that high-risk patients responded better to sorafenib, cisplatin and doxorubicin. Therefore, our gene signature provides the basis for the individualized application of ICIs and contributes to guide individualized chemotherapy and targeted therapy.

Undeniably, there are some drawbacks in our study. First, the exact mechanisms of modeling genes in hepatocarcinogenesis have not been explored, and thus, further studies are needed. Second, the risk signature was constructed and verified based on public databases. The 95% confidence intervals of the risk scores were somewhat wide in the Cox regression analyses, and the AUC for the ICGC cohort was larger than that for the TCGA cohort, which might be partly attributed to the divergent risk factors and pathogenesis of HCC in different regions. Therefore, a larger, multicenter cohort is demanded to further test the predictive capability of risk signature.

## Conclusions

In conclusion, we constructed the first BA-related gene signature, which presented a promising prediction performance and may be useful for making reasonable clinical decisions for HCC patients.

## Supplementary Information

Supplementary Figure S1.

Supplementary Figure S2.

Supplementary Figure S3.

Supplementary Figure S4.

Supplementary Figure S5.

Supplementary Figure S6.

Supplementary Figure S7.

Supplementary Legends.

Supplementary Tables.

## Data Availability

The data analyzed in the current study are freely available in the TCGA (https://portal.gdc.cancer.gov), ICGC (https://dcc.icgc.org/) and Gene Set Enrichment Analysis (http://www.gsea-msigdb.org/gsea/index.jsp) databases.

## Abbreviations

HCC: Hepatocellular carcinoma
BA: Bile acid
TCGA: The Cancer Genome Atlas
ICGC: International Cancer Genome Consortium
BAGs: Bile acid-related genes
GSEA: Gene Set Enrichment Analysis
LASSO: Least absolute shrinkage and selection operator
Log2FC: Log2 fold change
PCA: Principal component analysis
AUC: Area under the curve
GO: Gene Ontology
KEGG: Kyoto Encyclopedia of Genes and Genomes
ssGSEA: Single-sample gene set enrichment analysis
TMB: Tumor mutation burden
IC50: Semi-inhibitory concentration
RT–qPCR: Real-time quantitative PCR
BP: Biological process
CC: Cellular component
MF: Molecular function
DEGs: Differentially expressed genes
KM: Kaplan–Meier
aDCs: Activated dendritic cells
NK: Natural killer
ROC: Receiver operating characteristic
ERK1: Extracellular signal-regulated kinase 1
ICIs: Immune checkpoint inhibitors

## References

[1] Bray F, Ferlay J, Soerjomataram I (2018). Global cancer statistics 2018: GLOBOCAN estimates of incidence and mortality worldwide for 36 cancers in 185 countries. CA Cancer J. Clin..

[2] Zhang CH, Cheng Y, Zhang S (2022). Changing epidemiology of hepatocellular carcinoma in Asia. Liver Int..

[3] Llovet JM, Zucman-Rossi J, Pikarsky E (2016). Hepatocellular carcinoma. Nat .Rev. Dis. Primers.

[4] European Association for the Study of the Liver. Electronic address eee, European Association for the Study of the L. EASL clinical practice guidelines: Management of hepatocellular carcinoma. *J. Hepatol.* **69**(1), 182–236. 10.1016/j.jhep.2018.03.019 (2018). 10.1016/j.jhep.2018.03.019 29628281

[5] Shulpekova Y, Shirokova E, Zharkova M (2022). A recent ten-year perspective: Bile acid metabolism and signaling. Molecules.

[6] Fiorucci S, Biagioli M, Zampella A (2018). Bile acids activated receptors regulate innate immunity. Front. Immunol..

[7] Li M, Cai SY, Boyer JL (2017). Mechanisms of bile acid mediated inflammation in the liver. Mol. Aspects Med..

[8] Chiang JYL, Ferrell JM (2019). Bile Acids as metabolic regulators and nutrient sensors. Annu. Rev. Nutr..

[9] Jia W, Xie G, Jia W (2018). Bile acid-microbiota crosstalk in gastrointestinal inflammation and carcinogenesis. Nat. Rev. Gastroenterol. Hepatol..

[10] Xie G, Wang X, Huang F (2016). Dysregulated hepatic bile acids collaboratively promote liver carcinogenesis. Int. J. Cancer..

[11] Anderson CM, Stahl A (2013). SLC27 fatty acid transport proteins. Mol. Aspects Med..

[12] Gao Q, Zhang G, Zheng Y (2020). SLC27A5 deficiency activates NRF2/TXNRD1 pathway by increased lipid peroxidation in HCC. Cell Death Differ..

[13] Wang LX, Frey MR, Kohli R (2021). The role of FGF19 and MALRD1 in enterohepatic bile acid signaling. Front. Endocrinol. (Lausanne)..

[14] Raja A, Park I, Haq F (2019). FGF19-FGFR4 signaling in hepatocellular carcinoma. Cells.

[15] Tai DWM, Le TBU, Prawira A (2021). Targeted inhibition of FGF19/FGFR cascade improves antitumor immunity and response rate in hepatocellular carcinoma. Hepatol. Int..

[16] Mayakonda A, Lin DC, Assenov Y (2018). Maftools: Efficient and comprehensive analysis of somatic variants in cancer. Genome Res..

[17] Geeleher P, Cox N, Huang RS (2014). pRRophetic: An R package for prediction of clinical chemotherapeutic response from tumor gene expression levels. PLoS ONE.

[18] Kanehisa M, Goto S (2000). KEGG: Kyoto encyclopedia of genes and genomes. Nucleic Acids Res..

[19] Kanehisa M, Sato Y, Kawashima M (2016). KEGG as a reference resource for gene and protein annotation. Nucleic Acids Res..

[20] Choucair K, Morand S, Stanbery L (2020). TMB: A promising immune-response biomarker, and potential spearhead in advancing targeted therapy trials. Cancer Gene Ther..

[21] Lee M, Samstein RM, Valero C (2020). Tumor mutational burden as a predictive biomarker for checkpoint inhibitor immunotherapy. Hum. Vaccin. Immunother..

[22] Li X, Saha P, Li J (2016). Clues to the mechanism of cholesterol transfer from the structure of NPC1 middle lumenal domain bound to NPC2. Proc. Natl. Acad. Sci. USA.

[23] Geberhiwot T, Moro A, Dardis A (2018). Consensus clinical management guidelines for Niemann-Pick disease type C. Orphanet. J. Rare Dis..

[24] Rodriguez-Gil JL, Bianconi SE, Farhat N (2021). Hepatocellular carcinoma as a complication of Niemann-Pick disease type C1. Am. J. Med. Genet. A..

[25] Xu H, Diolintzi A, Storch J (2019). Fatty acid-binding proteins: Functional understanding and diagnostic implications. Curr. Opin. Clin. Nutr. Metab. Care..

[26] Lin CH, Chang HH, Lai CR (2022). Fatty acid binding protein 6 inhibition decreases cell cycle progression, migration and autophagy in bladder cancers. Int. J. Mol. Sci..

[27] Pai FC, Huang HW, Tsai YL (2021). Inhibition of FABP6 reduces tumor cell invasion and angiogenesis through the decrease in MMP-2 and VEGF in human glioblastoma cells. Cells.

[28] Ohmachi T, Inoue H, Mimori K (2006). Fatty acid binding protein 6 is overexpressed in colorectal cancer. Clin. Cancer Res..

[29] Yuan C, Yuan M, Chen M (2021). Prognostic implication of a novel metabolism-related gene signature in hepatocellular carcinoma. Front. Oncol..

[30] Anjum J, Mitra S, Das R (2022). A renewed concept on the MAPK signaling pathway in cancers: Polyphenols as a choice of therapeutics. Pharmacol Res..

[31] Cingolani F, Liu Y, Shen Y (2022). Redundant functions of ERK1 and ERK2 maintain mouse liver homeostasis through down-regulation of bile acid synthesis. Hepatol. Commun..

[32] Rezen T, Rozman D, Kovacs T (2022). The role of bile acids in carcinogenesis. Cell Mol. Life Sci..

[33] Song K-H, Li T, Owsley E (2009). Bile acids activate fibroblast growth factor 19 signaling in human hepatocytes to inhibit cholesterol 7α-hydroxylase gene expression. Hepatology.

[34] Wang LT, Liu KY, Chiou SS (2021). Phosphorylation of intestine-specific homeobox by ERK1 modulates oncogenic activity and sorafenib resistance. Cancer Lett..

[35] Ito Y, Sasaki Y, Horimoto M (1998). Activation of mitogen-activated protein kinases/extracellular signal-regulated kinases in human hepatocellular carcinoma. Hepatology.

[36] Li B, Zhou M, Wang J (2021). Suppressing ERK pathway impairs glycochenodeoxycholate-mediated survival and drug-resistance in hepatocellular carcinoma cells. Front. Oncol..

[37] Drury JE, Mindnich R, Penning TM (2010). Characterization of disease-related 5beta-reductase (AKR1D1) mutations reveals their potential to cause bile acid deficiency. J. Biol. Chem..

[38] Nikolaou N, Gathercole LL, Marchand L (2019). AKR1D1 is a novel regulator of metabolic phenotype in human hepatocytes and is dysregulated in non-alcoholic fatty liver disease. Metabolism.

[39] Gathercole LL, Nikolaou N, Harris SE (2022). AKR1D1 knockout mice develop a sex-dependent metabolic phenotype. J. Endocrinol..

[40] Lemonde HA, Custard EJ, Bouquet J (2003). Mutations in SRD5B1 (AKR1D1), the gene encoding delta(4)-3-oxosteroid 5beta-reductase, in hepatitis and liver failure in infancy. Gut.

[41] Nikolaou N, Gathercole LL, Kirkwood L (2019). AKR1D1 regulates glucocorticoid availability and glucocorticoid receptor activation in human hepatoma cells. J. Steroid Biochem. Mol. Biol..

[42] Zhu P, Feng R, Lu X (2021). Diagnostic and prognostic values of AKR1C3 and AKR1D1 in hepatocellular carcinoma. Aging (Albany NY)..

[43] Dai T, Ye L, Yu H (2021). Regulation network and prognostic significance of aldo-keto reductase (AKR) superfamily genes in hepatocellular carcinoma. J. Hepatocell. Carcinoma.

[44] Fu Y, Liu S, Zeng S (2019). From bench to bed: The tumor immune microenvironment and current immunotherapeutic strategies for hepatocellular carcinoma. J. Exp. Clin. Cancer Res..

[45] Biagioli M, Carino A (2019). Signaling from intestine to the host: How bile acids regulate intestinal and liver immunity. Handb. Exp. Pharmacol..

[46] Zhou J, Ding T, Pan W (2009). Increased intratumoral regulatory T cells are related to intratumoral macrophages and poor prognosis in hepatocellular carcinoma patients. Int. J. Cancer..

[47] Fu J, Xu D, Liu Z (2007). Increased regulatory T cells correlate with CD8 T-cell impairment and poor survival in hepatocellular carcinoma patients. Gastroenterology.

[48] Sun C, Sun HY, Xiao WH (2015). Natural killer cell dysfunction in hepatocellular carcinoma and NK cell-based immunotherapy. Acta Pharmacol. Sin..

[49] Li W, Huang X, Tong H (2012). Comparison of the regulation of beta-catenin signaling by type I, type II and type III interferons in hepatocellular carcinoma cells. PLoS ONE.

[50] Liu Z, Liu X, Liang J (2021). Immunotherapy for hepatocellular carcinoma: Current status and future prospects. Front. Immunol..

[51] Chalmers ZR, Connelly CF, Fabrizio D (2017). Analysis of 100,000 human cancer genomes reveals the landscape of tumor mutational burden. Genome Med..

[52] Stenzinger A, Allen JD, Maas J (2019). Tumor mutational burden standardization initiatives: Recommendations for consistent tumor mutational burden assessment in clinical samples to guide immunotherapy treatment decisions. Genes Chromosomes Cancer..

[53] Fumet JD, Truntzer C, Yarchoan M (2020). Tumour mutational burden as a biomarker for immunotherapy: Current data and emerging concepts. Eur. J. Cancer..

[54] Yarchoan M, Hopkins A, Jaffee EM (2017). Tumor mutational burden and response rate to PD-1 inhibition. N. Engl. J. Med..

[55] Geeleher P, Cox NJ, Huang RS (2014). Clinical drug response can be predicted using baseline gene expression levels and in vitro drug sensitivity in cell lines. Genome Biol..

[56] Ren W, Zuo S, Yang L (2022). Identification of a novel immune-related long noncoding RNA signature to predict the prognosis of bladder cancer. Sci. Rep..

[57] Chawla S, Rockstroh A, Lehman M (2022). Gene expression based inference of cancer drug sensitivity. Nat. Commun..

