# Supplementary figures and images for: A bile acid-related prognostic signature in hepatocellular carcinoma
Source: Sci Rep. 2022 Dec 26;12:22355. doi: 10.1038/s41598-022-26795-7 (PMC9792463; doi:10.1038/s41598-022-26795-7)

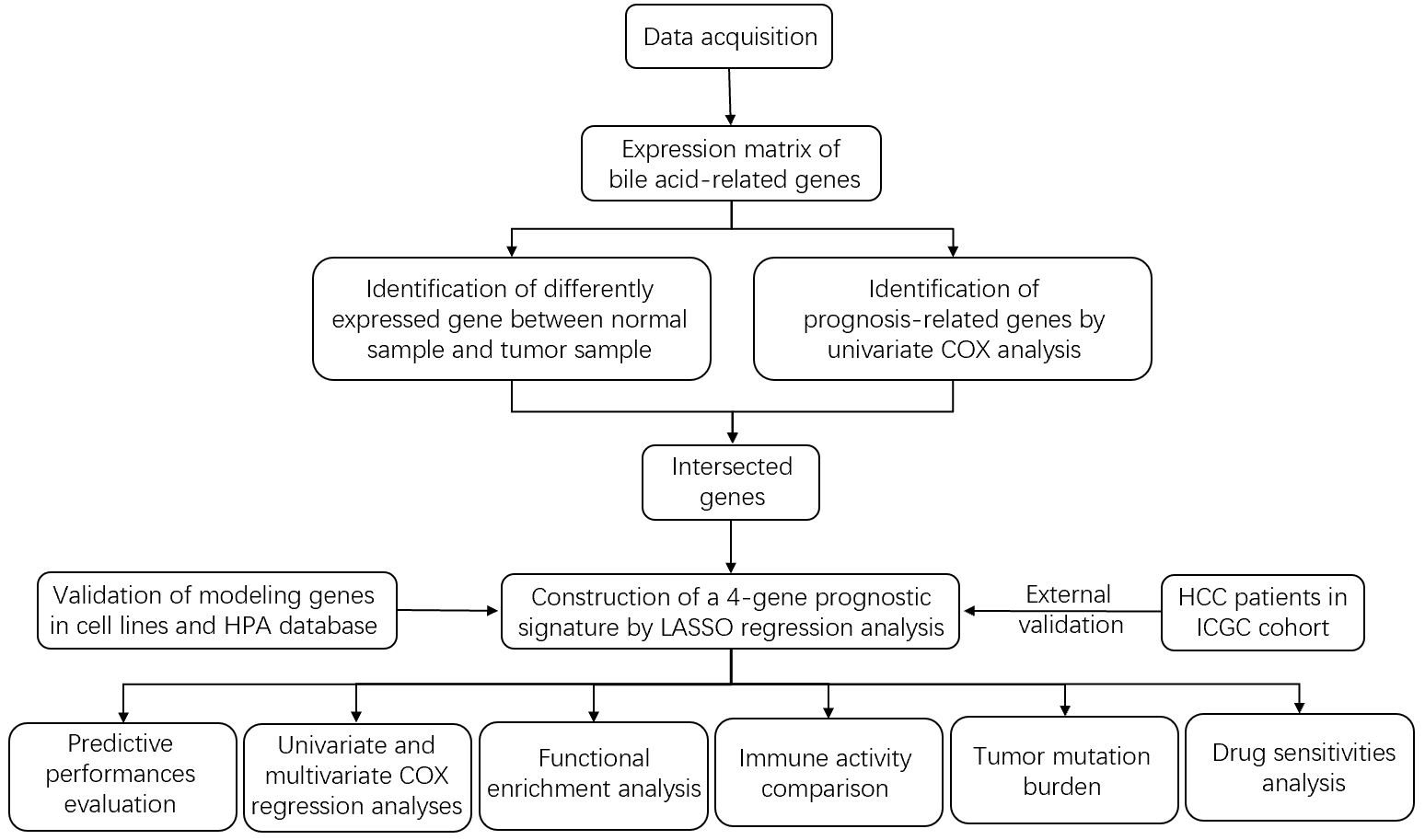

Supplement: Supplementary file 1 — Supplementary Figure S1. [file 41598_2022_26795_MOESM1_ESM.jpg]

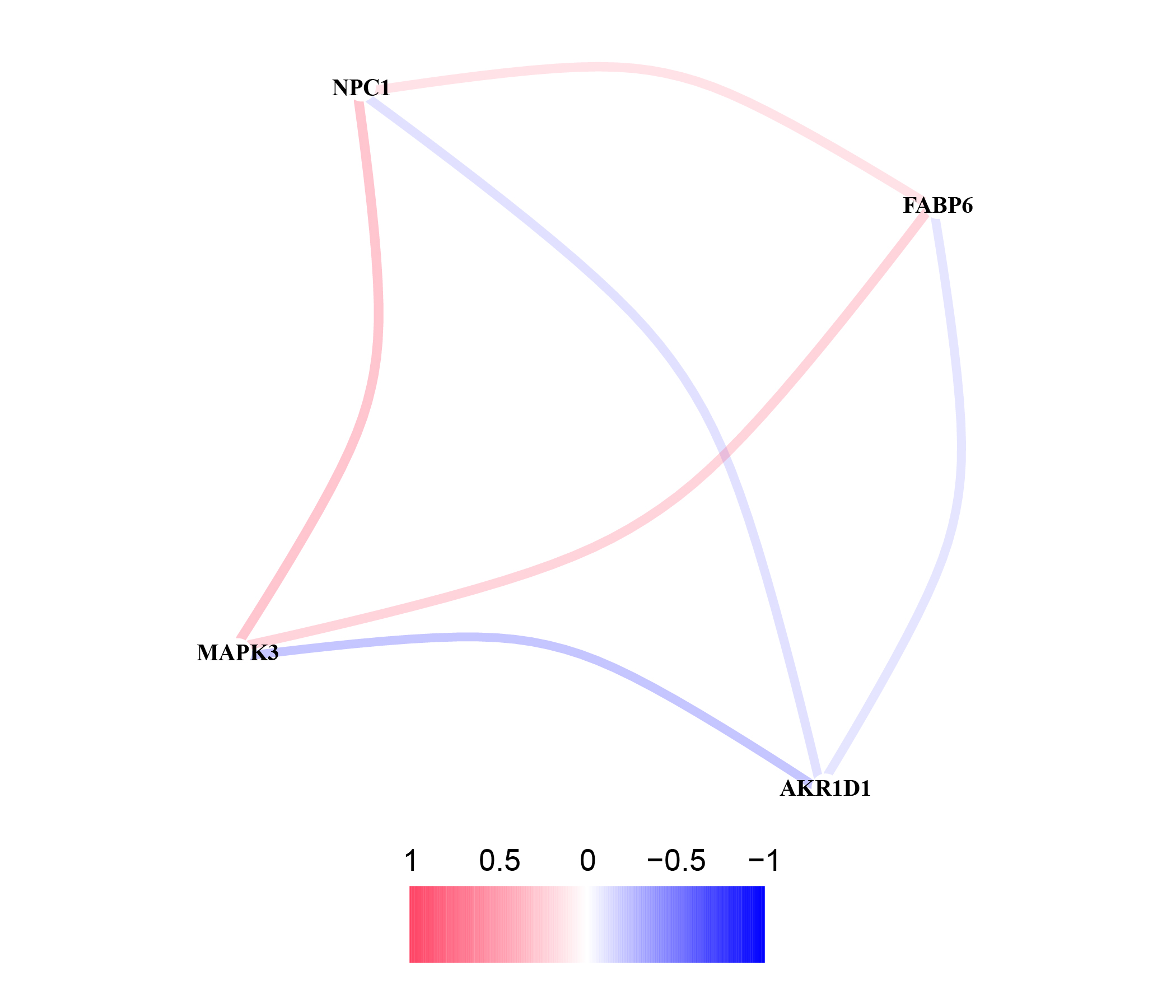

Supplement: Supplementary file 2 — Supplementary Figure S2. [file 41598_2022_26795_MOESM2_ESM.jpg]

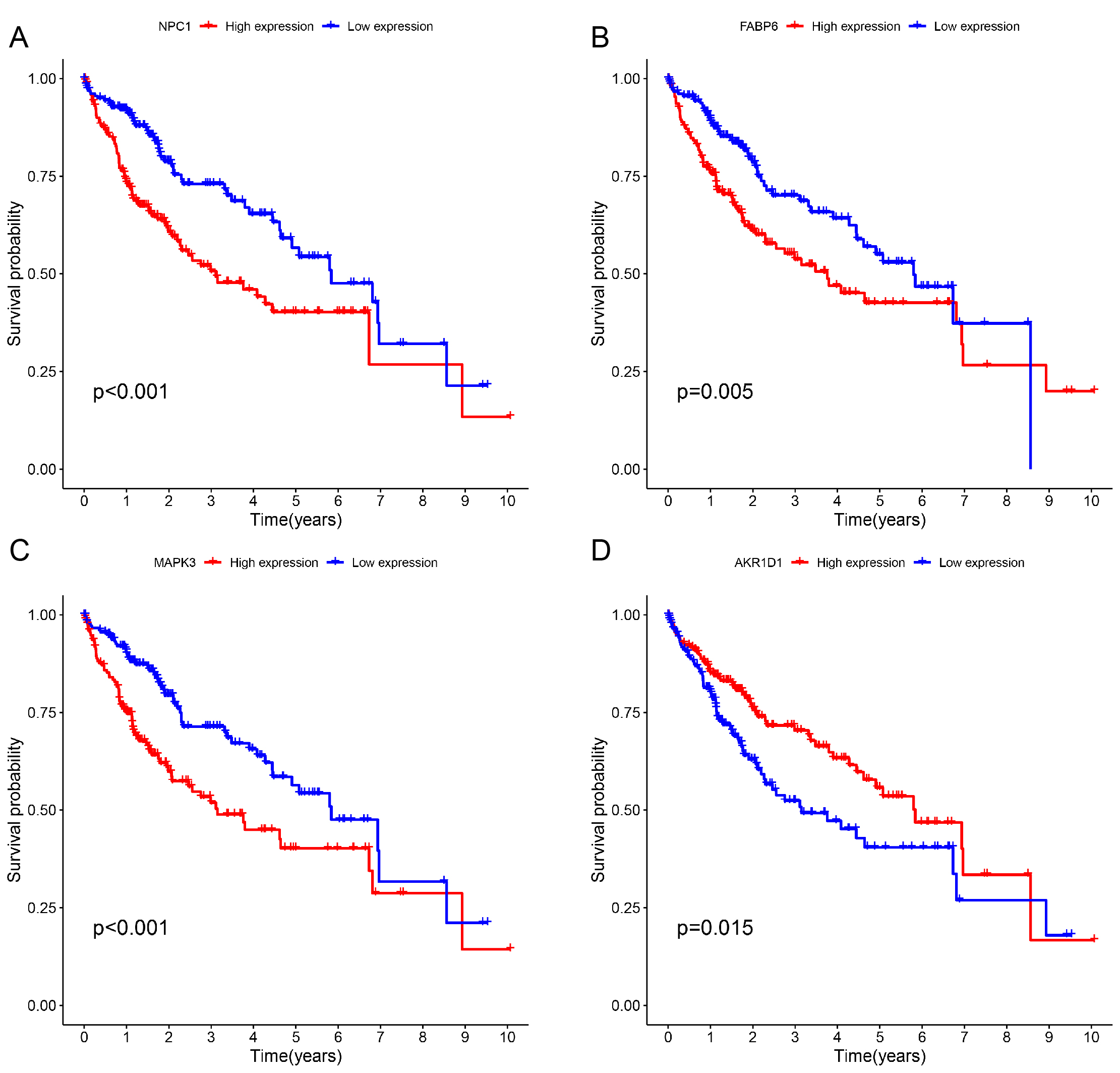

Supplement: Supplementary file 3 — Supplementary Figure S3. [file 41598_2022_26795_MOESM3_ESM.jpg]

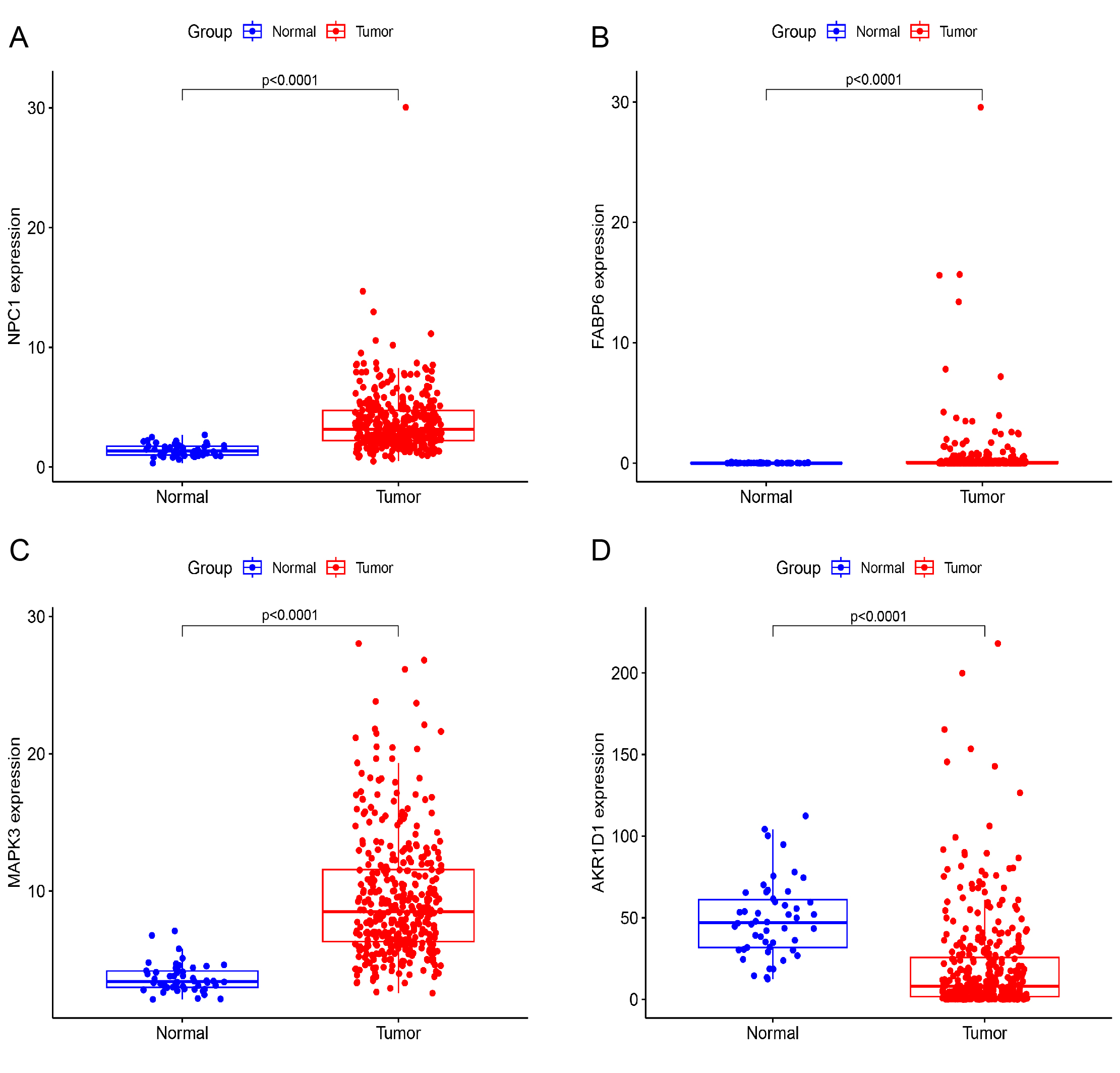

Supplement: Supplementary file 4 — Supplementary Figure S4. [file 41598_2022_26795_MOESM4_ESM.jpg]

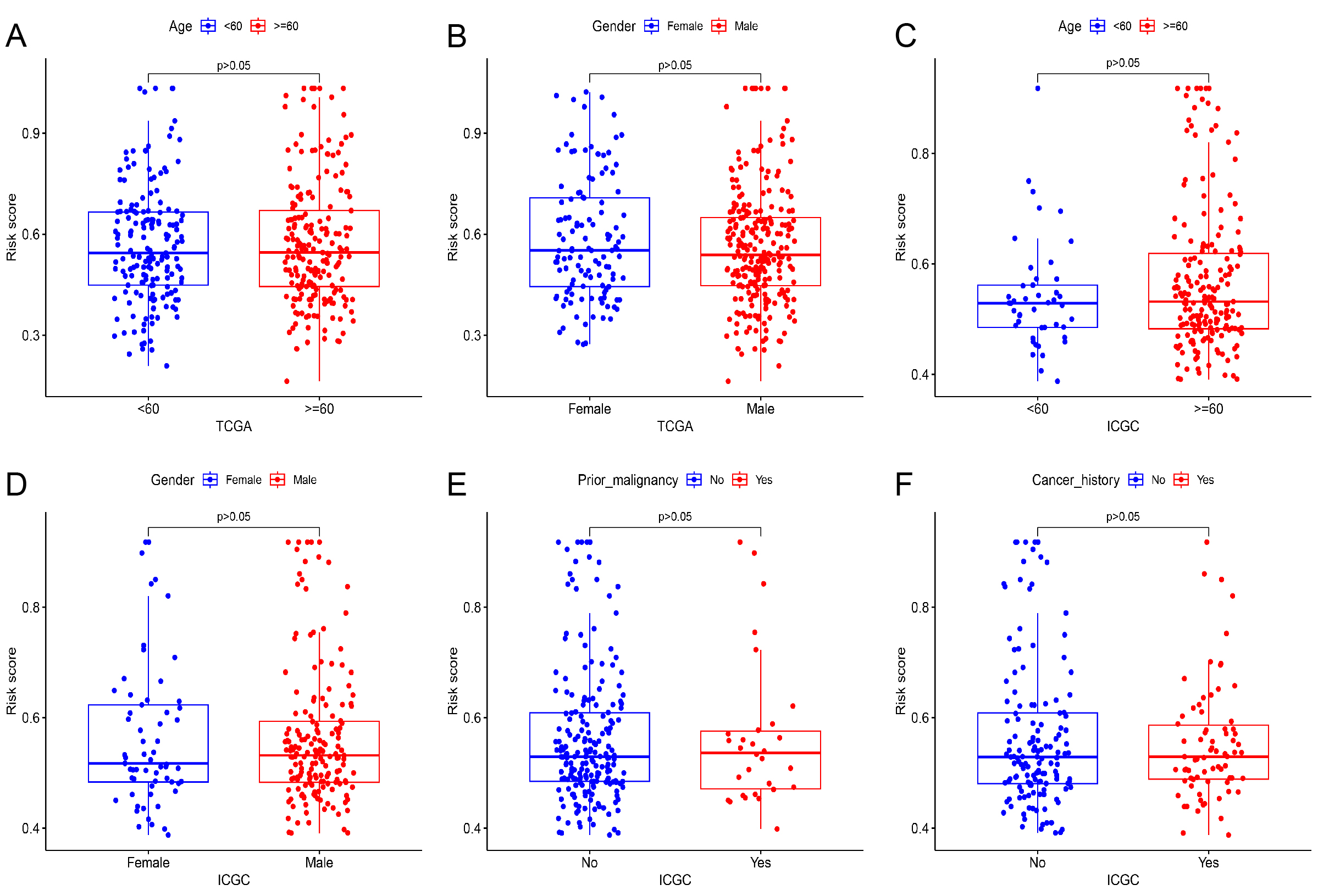

Supplement: Supplementary file 5 — Supplementary Figure S5. [file 41598_2022_26795_MOESM5_ESM.jpg]

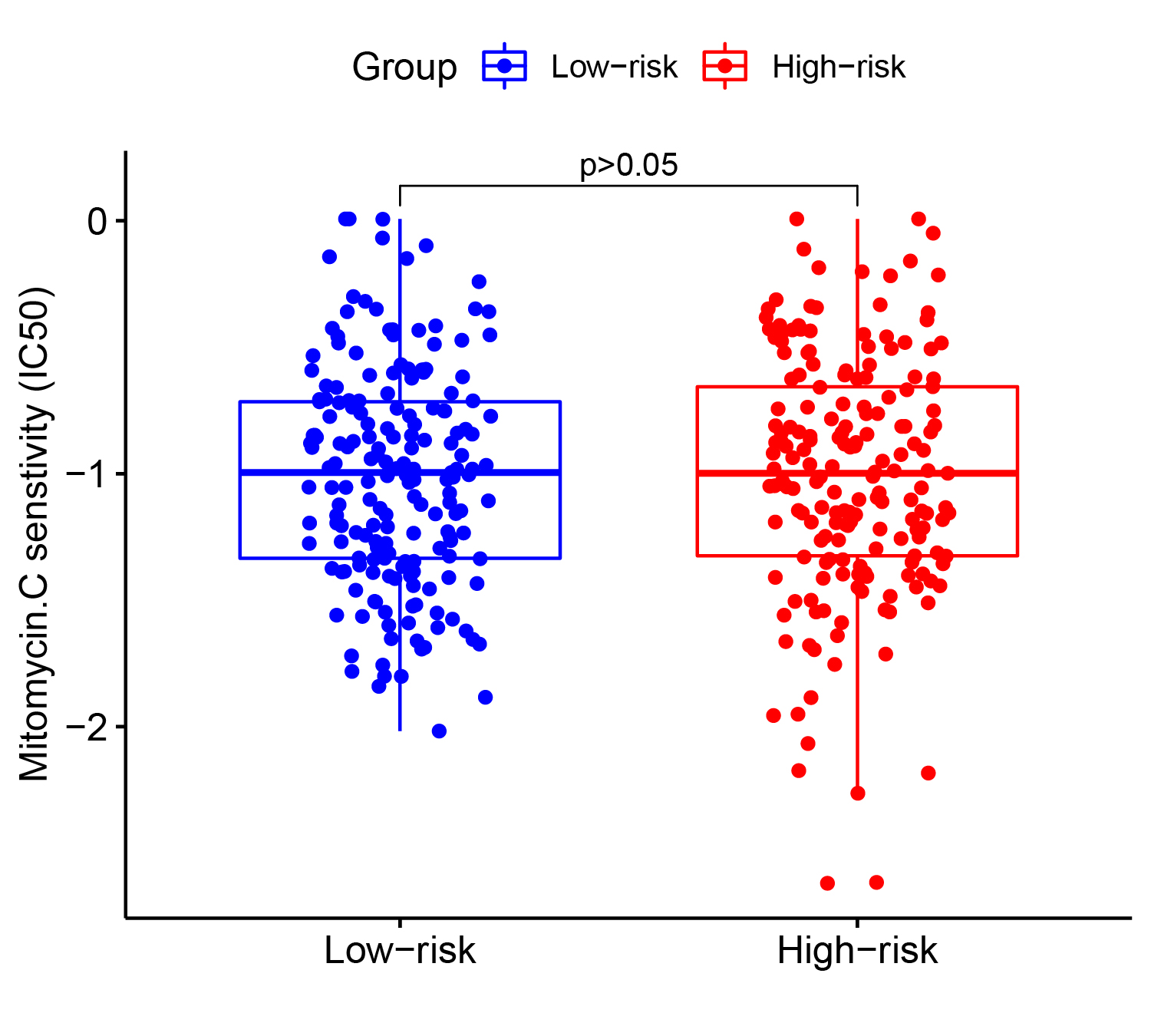

Supplement: Supplementary file 6 — Supplementary Figure S6. [file 41598_2022_26795_MOESM6_ESM.jpg]

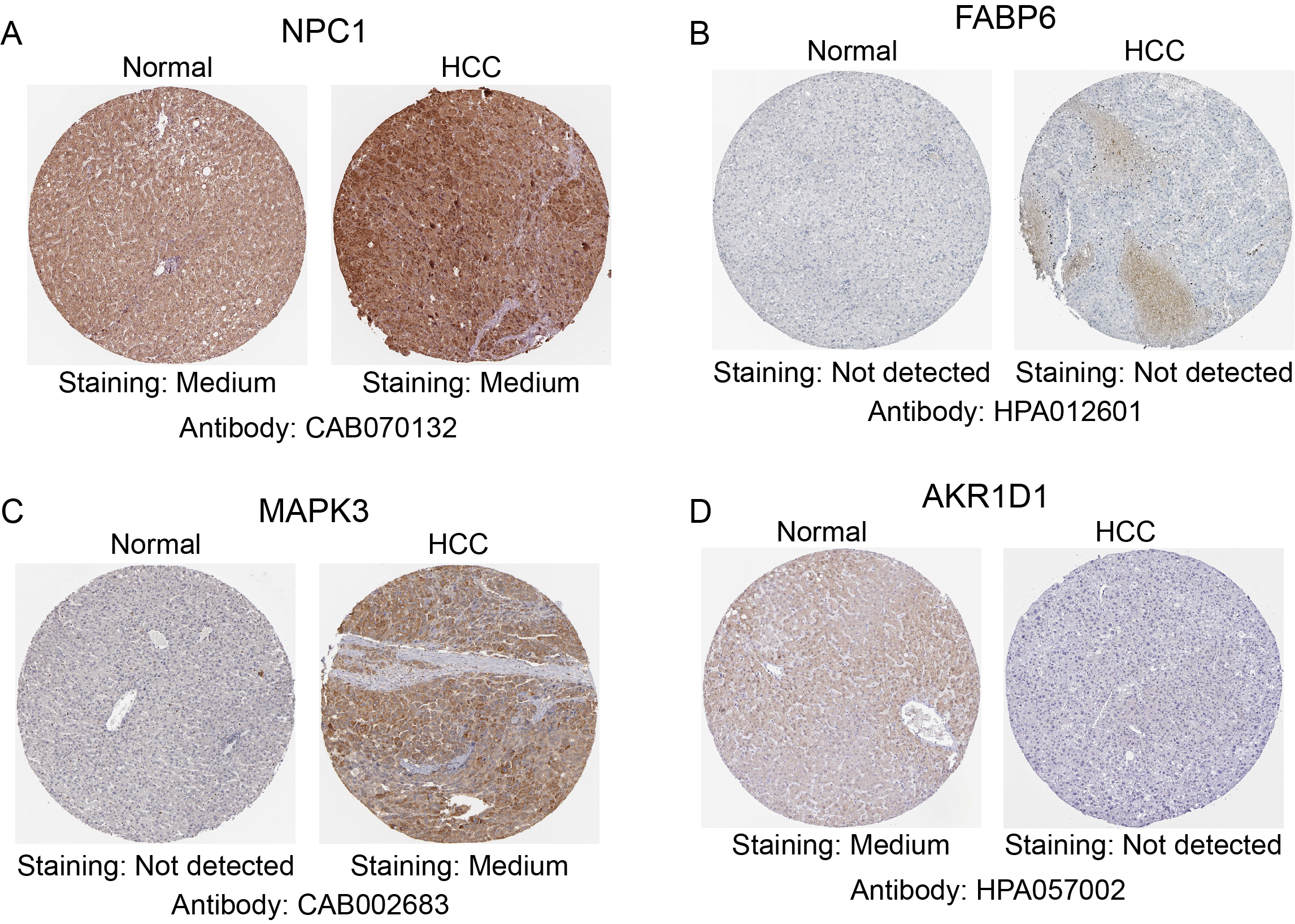

Supplement: Supplementary file 7 — Supplementary Figure S7. [file 41598_2022_26795_MOESM7_ESM.jpg]
