# Supplementary material for: A bile acid-related prognostic signature in hepatocellular carcinoma
Source: Sci Rep. 2022 Dec 26;12:22355. doi: 10.1038/s41598-022-26795-7 (PMC9792463; doi:10.1038/s41598-022-26795-7)
Supplement: Supplementary file 8 — Supplementary Legends. [file 41598_2022_26795_MOESM8_ESM.docx]

Supporting legends

Fig. S1 The analysis flow of this study. The expression matrix of bile acid-related genes was extracted from TCGA database. Differentially expressed prognostic genes were obtained by intersecting the differentially expressed genes with the prognostic genes, which were further analyzed by LASSO regression analysis. Finally, 4 differentially expressed prognostic genes were obtained to construct a prognostic signature. Patient risk score was calculated based on the prognostic signature. Then, patients were classified into high- and low-risk groups according to their median scores. We evaluated and validated the predictive performance of the risk signature in TCGA and ICGC cohort, respectively. We further conducted functional enrichment analysis, immune activity comparison, tumor mutation burden and drug sensitivities analysis in different risk groups. In addition, the protein and mRNA levels of the modeling genes were validated in the Human Protein Atlas database and our cell lines, respectively.

Fig. S2 Correlation network of modeling genes. Red indicates positive correlation, and blue indicates negative correlation.

Fig. S3 The relationships of modeling gene expression levels and overall survival were evaluated. (A) NPC1, (B) FABP6 and (C) MAPK3 were risk factors for overall survival, while (D) AKR1D1 were protective factors.

Fig. S4 The expression levels of modeling genes in normal and HCC samples. (A) NPC1, (B) FABP6 and (C) MAPK3 were upregulated, while (D) AKR1D1 were downregulated in the tumor samples.

Fig. S5 Risk score-related clinicopathological features. (A-B) Age and gender in TCGA cohort. (C-F) Age, gender, prior malignancy and cancer history in ICGC cohort.

Fig. S6 The sensitivity comparisons for mitomycin based on risk grouping.

Fig. S7 Immunohistochemical images of modeling genes in normal and HCC tissues. (A) NPC1, (B) FABP6, (C) MAPK3 and (D) AKR1D1.
